# Délégation des tâches dans le domaine de la planification familiale au Burkina Faso: qualité des services offerts par le délégataire

**DOI:** 10.11604/pamj.2020.36.69.18091

**Published:** 2020-06-05

**Authors:** Souleymane Kaboré, Roland Sanou, Boureima Baillou, Isabelle Zongo, Alidou Zongo, Elizabeth Kondé, André Yolland Ky, Ida Salou Kagoné, George Coulibaly, Djénéba Sanon Ouédraogo, Robert Karama

**Affiliations:** 1 Direction Régionale de la Santé du Centre Est, Burkina Faso,; 2 District Sanitaire de Tougan, Tougan, Burkina Faso,; 3 Direction Régionale de la Santé de la Boucle du Mouhoun, Mouhoun, Burkina Faso,; 4 Direction de la Santé de la Famille, Burkina Faso,; 5 Secrétariat Technique Dividende Démographique du Ministère de la santé, Burkina Faso,; 6 Marie Stoppes Burkina Faso,; 7 Fonds des Nations Unies pour la Population, Burkina Faso,; 8 Secrétariat Général du Ministère de la Santé, Burkina Faso

**Keywords:** Délégation des tâches, qualité, planification familiale, délégant, délégataire, Task sharing, quality, family planning, delegators, delegatees

## Abstract

**Introduction:**

pour améliorer l’accessibilité des méthodes contraceptives au Burkina Faso, il a été initié un projet pilote de transfert des compétences de l’offre du DIU et de l’implant aux agents de santé première ligne (APL) ainsi que l’offre des méthodes contraceptives injectables aux agents de santé communautaire (ASC) dans 20 centres de santé du district sanitaire de Tougan. La présente étude visait à apprécier la qualité des services de planification familiale (PF) offerts par ces délégataires (APL et ASC).

**Méthodes:**

il s’est agi d’une étude transversale à visée descriptive et analytique. La collecte des données a combiné les méthodes quantitatives et qualitatives. Elle a porté sur l’ensemble des 20 centres de santé de la zone d’intervention du projet et sur tous les prestataires (54) impliqués dans l’offre des produits contraceptifs (délégants et délégataires). Dix-neuf (19) bénéficiaires dont 10 nouvelles utilisatrices d’une méthode contraceptive ont été interviewées. Les techniques de collecte étaient constituées de l’observation des prestations de services de PF et de l’environnement de travail, de la revue documentaire et des entretiens individuels. Les données ont été analysées à l’aide du logiciel Epi info 7 et Open Epi version 3.01. Le test du Chi carré et le test t de Student ont été utilisés pour déterminer s’il y existait une différence significative entre la qualité des services de PF offerts par les délégants et celle des délégataires.

**Résultats:**

le score de qualité générale du service de PF de la zone d’intervention était de 73% chez les délégataires contre 69% chez les délégants. Il n’y avait pas de différence statistiquement significative entre ces scores. Par contre, Il existait une différence statistiquement significative entre le score de qualité des agents de santé communautaire (75,8 %) et celui des délégants (87,5 %) en matière counseling (P <0.05). Il en était de même pour le score de qualité en matière de détermination des critères d’éligibilité des implants où la qualité chez les APL semble supérieure à celle des délégants: 79% pour les délégataires, 64% pour les délégants.

**Conclusion:**

cette expérience a eu l’avantage d’améliorer la couverture géographique de l’offre des méthodes contraceptives de longue durée d’action. Sous certaines conditions (renforcement des compétences, suivi, coaching), il est bien possible d’étendre l’offre des méthodes contraceptives de longues durées aux APL ainsi que celle des injectables aux ASC tout en conservant un niveau de qualité des services PF satisfaisant.

## Abstract (en)

**Introduction:**

we conducted a pilot study for transferring skills for intrauterine device (IUD) insertion and implants to primary health care workers (PHCWs) as well as to provide injectable contraceptives to community health workers (CHWs) in 20 Health Centers in the Tougan Health District. This was aimed to increase access to contraceptive methods in Burkina Faso. Moreover, the purpose of this study was to assess the quality of family planning (PF) services offered by these delegated (PHCWs and CHWs).

**Methods:**

we conducted a cross-sectional, descriptive, analytical study. Data collection was based on quantitative and qualitative methods. It included the 20 health centers in the study area and all providers (54) involved in contraceptive product supply (delegators and delegatees). Nineteen (19) recipients including 10 new contraceptive users were interviewed. The method of data collection included the observation of FP services and of the working environment, document review and individual interviews. Data were analyzed using Epi info 7 and Open Epi version 3.01. software. Chi-square test and Student’s t-test were used to determine whether there was significant difference between the quality of PF services offered by the delegators and that of the delegatees.

**Results:**

PF service quality score in the study area was 73% for the delegators’ vs 69% for the delegates. There was no statistically significant difference between these scores. However, there was a statistically significant difference between the quality score of community health workers (75.8%) and the quality score of counselors (delegators; 87.5%) (P <0.05). It was the same for quality score determining who was eligible for implants. The quality score of PHCWs was higher than that of delegators: 79% for delegators, 64% for delegatees.

**Conclusion:**

this purpose of this study was to improve the geographic coverage for long-acting contraceptive methods. Under certain conditions (skills-building, monitoring, coaching), it is possible to extend the transferring of skills for long-acting contraceptive methods to PHCWs as well as the provision of injectable contraceptives to CHWs, while maintaining a satisfactory level of FP service quality.

## Introduction

L’offre des services de planification familiale (PF) pourrait permettre de réduire d’environ 30%, le nombre de décès maternels dans le monde. Malheureusement la faible couverture géographique des méthodes contraceptives modernes en raison des pénuries en personnel de santé limite l’accès aux produits contraceptifs [1]. En effet, malgré la mise en œuvre de projets pilotes de transfert des compétences dans le domaine de la PF dans certains pays en Afrique et en Asie [2-5], les documents de politique et normes en matière de santé de la reproduction de nombreux pays restreignent l’offre des méthodes contraceptives de longues durées d’action aux sages-femmes et aux médecins [6-8]. Au Burkina Faso, bien que le ratio de mortalité maternelle soit un des plus élevé au monde et que les besoins non satisfaits en matière de la contraception restent très alarmants (19,4% des femmes en âge de procréer) [9], le document de Politique et Normes en matière de la santé de la reproduction n’autorisait pas les agents de première ligne (infirmier(è)s breveté(e)s (IB), accoucheuses auxiliaires (AA), accoucheuses brevetées (AB) et agents itinérants de santé (AIS)) à offrir ni les méthodes de longues durées d’action, ni les injectables [10].

Aussi, le ministère de la Santé burkinabè avec l’appui de ses partenaires techniques et financiers a élaboré un Plan National d’Accélération de la Planification Familiale couvrant la période 2017-2020 avec pour objectif d’atteindre 32% de prévalence contraceptive en 2020 [8]. Pour contribuer à l’atteinte de ce résultat, il a été initié dans le district sanitaire de Dandé (région des Hauts Bassins) et celui de Tougan (région de la Boucle du Mouhoun) un projet pilote d’expérimentation de la délégation des tâches dans le domaine de la planification familiale (projet DT). Prévu pour durer 24 mois, ce projet consistait d’une part à autoriser les agents de première ligne (APL) à offrir les méthodes contraceptives de longue durée d’action (implant et DIU) dans les centres de santé publics et d’autre part à autoriser les agents de santé communautaire (ASC) à offrir la pilule et les contraceptifs injectables. Vingt (20) formations sanitaires ont été retenues pour la mise en œuvre de ce projet dans le district sanitaire de Tougan. La délégation des tâches dans le domaine de l’offre des méthodes contraceptives modernes est une stratégie de repositionnement de la PF recommandée par l’OMS. Elle est en expérimentation dans plusieurs pays africains avec des résultats forts encourageants [11,12]. Toutefois, il est préconisé de maintenir la qualité et la sécurité des services fournis lors de sa mise en œuvre [1]. C’est à ce titre qu’avec le financement de Marie Stoppes Burkina, la présente étude a été réalisée pour apprécier le niveau de qualité des services de PF offerts par les délégataires afin de documenter l’intervention et de proposer des améliorations.

## Méthodes

**Cadre de l’étude:** l’étude a été réalisée dans le district sanitaire de Tougan, une des zones de mise en œuvre du projet DT. Situé au nord-ouest du Burkina Faso, à 230 km de la capitale, ce district comptait en 2017, 46 centres de santé et un hôpital de district. Il est peuplé par une population de 273 392 habitants (dont 60 366 femmes en âge de procréer) répartie dans 176 villages et huit communes. Chaque village disposait d’au moins deux agents de santé communautaires. Sur les 46 centres de santé, seuls 13 disposaient d’une sage-femme. Le district comptait un effectif cumulé de 252 agents de santé dont 66 IDE, 34 IB, 5 AB, 32 AIS, 42 sages-femmes (SFE). Les taux d’utilisation des méthodes contraceptives du district étaient de 23,88% en 2015 et de 20,49% en 2016 [13]. Dans le district sanitaire de Tougan, l’intervention était mise en œuvre sous la forme d’un projet pilote dans les communes de Kiembara et de Tougan.

### Description de l’intervention

**Offre des méthodes contraceptives avant l’intervention**

**Implication du personnel de santé selon la qualification dans l’offre des méthodes contraceptives modernes:** avant l’intervention DT et conformément au PNP, seules les sages-femmes, les infirmiers d’Etat, les attachés de santé et les médecins formés étaient autorisés à offrir le dispositif intra-utérin (DIU), les implants et les injectables. Tout le personnel de santé (AIS, AA, IB, AB, SFE/ME, IDE, attachés et médecins) était habilité à offrir les contraceptifs oraux. Outre la prescription des produits contraceptifs, les AIS et les AA n’étaient pas autorisés à prendre en charge les effets secondaires causés par les méthodes contraceptives. Quant aux agents de santé communautaire, ils étaient autorisés à réaliser uniquement le renouvellement des condoms, des spermicides et des pilules.

**Organisation de l’offre des méthodes contraceptives dans les centres de santé et les villages:** dans les centres de santé ne disposant pas de personnel habilité à initier le DIU ou l’implant le personnel de santé pouvait quand même en faire la promotion. Il en était de même pour les agents de santé dans les villages. Cependant ils devaient référer la cliente dans une structure de soins qui dispose du personnel autorisé et compétent pour l’offre de ladite méthode. Par moment et avec le financement du Fonds des nations unies pour la population, le personnel rassemblait un groupe de clientes désireuses de méthodes de longues durées d’action et faisait appel aux sages-femmes de l’hôpital du district pour l’offre de ces méthodes contraceptives sur site. Cette organisation a l’inconvénient de réduire l’accessibilité et l’adhésion des populations aux méthodes de longues durées d’action. Elle entame également le principe du choix volontaire et indépendant de la méthode contraceptive par la cliente qui n’a pas accès à la méthode de son choix. A côté de cette organisation classique, certains agents de santé communautaire étaient autorisés à réaliser dans le cadre d’un projet pilote (Health Policy Project) la distribution à base communautaire de contraceptifs oraux dans les villages [10].

**Approvisionnement des CSPS en produits contraceptifs:** l’approvisionnement des centres de santé en produits contraceptifs se faisait au niveau du dépôt répartiteur du district qui les reçoit du central d’achat des médicaments génériques (CAMEG). Un suivi hebdomadaire du stock des produits contraceptifs des centres de santé était assuré par les pharmaciens du district, de la direction régionale de la santé et de la direction de la santé de la famille.

### Changements apportés par l’intervention

**Transfert de compétence de l’offre des méthodes contraceptives modernes aux prestataires de 1ère ligne et aux agents de santé communautaire:** le projet DT a expérimenté d’une part l’offre du DIU, de l’implant et du contraceptif injectable par les prestataires de soins de 1ère ligne qui n’en étaient pas autorisés (AIS, AA, IB, AB) et d’autre part l’offre des injectables et des pilules par les agents de santé communautaire en communauté.

**Renforcement des compétences des délégataires:** ces nouveaux prestataires (délégataires) ont été formés en PF clinique pendant une dizaine de jours. Au cours de chaque session de formation les délégataires ont bénéficié d’abord d’un cours théorique sur le counseling, le bilan clinique, les effets secondaires, l’administration de la méthode, la prévention des infections et la gestion des déchets. Ensuite, ils ont réalisé des jeux de rôles, des prestations sur mannequin et des pratiques à temps réel sur des clientes. Pour valider cette formation et certifier que le délégataire était apte à offrir la nouvelle méthode contraceptive, il devait offrir de façon correcte et sous la supervision d’un mentor la méthode contraceptive à dix (10) clientes au moins. Un suivi post formation a été réalisé sur site et de façon trimestrielle par l’équipe projet (constituée des agents terrain de l’ONG Marie-stoppes Burkina Faso et de l’Association burkinabè du bien-être familiale), l’Equipe cadre du district de Tougan et la direction régionale de la santé de la Boucle du Mouhoun.

**Nouvelle organisation de l’offre des méthodes contraceptives:** les centres de santé de la zone d’intervention du projet ont été renforcés en équipement et en particulier en produits contraceptifs. Après la validation de la compétence des délégataires agents de santé, ils ont été invités à offrir au même titre que les sages-femmes le DIU, les implants et les injectables. Quant aux agents de santé communautaire, ils offraient les injectables et les pilules dans en communauté. Ils s’approvisionnaient en produits contraceptifs auprès des centres de santé et les revendaient aux bénéficiaires au même prix qu’au centre de santé. Ces agents de santé communautaire bénéficiaient d’une rétribution mensuelle de vingt mille (20 000) francs CFA (environ 33 dollars US) octroyés par l’Etat burkinabè.

**Type d’étude:** il s’est agi d’une étude transversale à visée descriptive et analytique. Elle a combiné les méthodes quantitatives et qualitatives.

**Période d’étude:** l’étude s’est étalée sur une période cinq mois (novembre à mars) avec une collecte des données de 5 jours allant du 13 au 17 décembre 2017.

**Population cible:** la population cible de l’étude était constituée des femmes en âge de procréer du district sanitaire de Tougan (62 137 bénéficiaires), de tous les prestataires de soins et des agents de santé communautaires du district. La collecte des données a concerné les 20 formations sanitaires (18 centres de santé ruraux, un centre de santé urbain et 1 centre médical) réparties dans les deux communes d’intervention du projet DT (Tougan et Kiembara).

**Echantillonnage:** l’enquête quantitative a porté de façon exhaustive, sur l’ensemble des 20 centres de santé de la zone d’intervention du projet DT du district sanitaire de Tougan. Dans chaque centre de santé, elle s’est adressée à tous les prestataires de soins chargés de l’offre des méthodes contraceptives (délégataires et délégants) ainsi qu’à deux agents de santé communautaires. Un des deux agents de santé communautaire a été désigné dans un village situé à plus de 5 km du centre de santé. Les participantes à l’enquête qualitative ont été identifiées parmi les femmes ayant bénéficié d’une méthode contraceptive auprès des délégataires. Au total, 20 bénéficiaires (dont 50% de nouvelles utilisatrices) ont été retenus: 10 bénéficiaires d’une méthode de longue durée d’action (5 DIU et 5 implants) et 10 bénéficiaires d’une méthode contraceptive offerte par les agents de santé communautaire (5 injectables et 5 orales). Cinq bénéficiaires ont été désignés dans un village situé à plus de 5 km du CSPS. Pour la sélection des bénéficiaires, il a été utilisé comme base de sondage, le registre de PF du centre de santé et celui des agents de santé communautaire. Après avoir identifié les bénéficiaires ayant reçu une méthode contraceptive moderne auprès des délégataires, l’on les a regroupés par méthode, leur a affecté un numéro d’ordre (1 à n) et a calculé un pas de sondage (n/5). La première bénéficiaire a été choisie entre 1 et n/5, puis les autres bénéficiaires ont été choisis en ajoutant 4 fois le pas de sondage au numéro de la première bénéficiaire.

### Critères d’inclusion

Ont été inclus dans cette étude: **prestataires**, tous les prestataires de soins travaillant dans un des centres de santé de la zone d’intervention du projet DT. **Agents de santé communautaire**: les agents de santé communautaire exerçant dans un village de la zone d’intervention du projet DT et formés à l’offre des méthodes contraceptives dans le cadre du projet. **Utilisatrices** (bénéficiaires): les utilisatrices de méthodes contraceptives résident dans la zone d’intervention et ayant bénéficié d’une méthode contraceptive auprès des délégataires.

**Techniques et outils de collecte des données:** les techniques de collecte suivantes ont été utilisées: la revue documentaire; l’observation non participante des prestations de services de PF; l’observation de l’environnement et des équipements de travail; l’entretien individuel avec les utilisatrices (utilisatrices des méthodes contraceptives offertes par les délégataires); l’entretien individuel avec les prestataires (délégataires et délégants); et l’entretien individuel avec les agents de santé communautaire.

Les outils de collecte utilisés étaient:

**La grille de revue documentaire** composée de 72 questions réparties en trois rubriques (sage-femme et infirmier d’Etat; agent de 1ère ligne et agents de santé communautaire) et neuf sous rubriques constituées comme suit: nombre de DIU, d’implants, de pilules, de méthodes injectables, de complications/incidents pour la pose de DIU, de complications/incidents pour l’insertion du Jadelle, de complications/incidents après injection, d’accidents d’exposition au sang ou produit biologique.

**Deux guides d’observation:** Le 1er guide d’observation était constitué de 57 questions réparties en deux volets: un volet CSPS et un volet agents de santé communautaire. La partie CSPS est répartie en sept rubriques que sont: disponibilité des documents, normes et outils de surveillance; organisation du service de PF composée des éléments suivants: description des postes, répartition des tâches, permanence du service PF, local adapté (confidentialité, propreté), disponibilité des supports pour le counseling (boite à image, affiche, échantillon de tous les produits contraceptifs), dispositif de recherche des perdus de vue en PF, intégration de la PF aux autres services de PF; Disponibilité des intrants: produits contraceptifs, instruments, équipements et outils de collecte; Offre de la méthode contraceptive; application des prix de vente et circuit d’approvisionnement des produits contraceptifs: circuit, prix de vente, fiche de stock, bon de commande, gratuité des consommables; gestion des déchets et contrôle et prévention des infections: poubelle adaptée, gans, dispositif de pré et désinfection, boites à tranchant, incinérateur, protocole de gestion des accidents d’exposition aux produits biologiques; promotion de la PF et supervisons des prestataires. Le volet agents de santé communautaire du guide d’observation est réparti en quatre rubriques que sont: approvisionnement des intrants des agents de santé communautaire: circuit d’approvisionnement, disponibilité des produits contraceptifs et intrants, boite à aiguilles; offre de la méthode contraceptive; suivi et Surveillance: rapport, supervision; Promotion de la PF: outils de sensibilisation, programme de sensibilisation. Le deuxième guide d’observation étaient de quatre volets: un volet DIU, un volet implant, un volet injectable et un volet pilule. Pour les agents de santé communautaire, seuls les volets injectables et pilule leur étaient administrés. Chacun de ces volets était constitué de 19 questions réparties en six rubriques que sont: maitrise de l’accueil et counseling; maitrise du bilan de la cliente; maitrise de la technique d’administration du produit contraceptif; maitrise de la gestion des déchets et le contrôle et prévention des infections; maitrise du counseling post-insertion; remplissage des supports.

**Le questionnaire semi structuré destiné aux prestataires:** il était destiné à mesurer le niveau de connaissance des prestataires (délégataires et délégants) et était constitué des rubriques suivantes: connaissance sur le counseling: avantages, définition des catégories d’utilisatrices, exploration du VIH et IST; connaissance sur la réalisation du bilan: détermination d’une éventuelle grossesse; examen clinique; connaissance de la technique d’administration du produit contraceptif; connaissance sur la gestion des déchets, le contrôle et la prévention des infections; Connaissance sur la gestion des problèmes courants: effet secondaires, conduite à tenir en cas d’oubli d’une pilule, vérification de la présence du DIU; la fréquence de l’offre des méthodes contraceptives par le prestataire.

**Le questionnaire semi structuré destiné aux bénéficiaires:** il s’est agi d’un guide d’entretien avec les utilisatrices des méthodes contraceptives ayant bénéficié d’une prestation auprès des délégataires. Il visait le recueil de l’expérience de la cliente (de son vécu) lors les soins contraceptifs, en vue de la réalisation d’une triangulation des informations collectées auprès des délégataires. Il était constitué de sept rubriques que sont: informations générales relatives à la gestité, la parité, le type de méthode choisie, statut (nouvelle ou ancienne); mode de recrutement: lieu, circonstance; expérience sur l’accueil et le counseling: empathie, entretien, informations clées sur la méthode contraceptive y compris le VIH et IST, confidentialité, choix éclairé et indépendante de la méthode; expérience relative à l’administration du produit contraceptif: prix, bilan, utilisation d’anesthésique, site d’administration, prévention des infections, douleur, conseils post administration; Opinions sur la délégation des tâches.

### Conditions de validation des outils et de la collecte des données

**Conditions de validation des outils:** pour l’élaboration des outils de collecte, les documents suivants ont été utilisés: - directives pour l’évaluation du prestataire compétents [14]; everybody’s business, strengthening health systems to improve health outcomes [15]; planification familiale: Manuel de formation à l’intention des prestataires de service du monde entier [16]; standards for improving quality of maternal and newborn care in health facilities [17]. Après cette élaboration des outils, un comité de pilotage qui est constitué de deux médecins de santé publique, deux infirmiers spécialisés en épidémiologie, un infirmier spécialisé en santé publique a passé en revue chaque question et procédé à leur validation. Les outils ont enfin été testés dans un centre de santé du district sanitaire de Dédougou pendant la formation des enquêteurs. Après ce prétest, les différents amendements ont été intégrés aux outils avant leur utilisation sur le terrain.

**Conditions de validation de la collecte des données** La collecte de données quantitative a été réalisée à l’aide des questionnaires prestataire, agents de santé communautaire et d’un guide d’observation par quatre enquêteurs répartis en deux binômes composés d’une femme au moins. Les enquêteurs avaient au moins sept ans d’expérience et le profil suivant: trois sages-femmes et un infirmier spécialisé en soins obstétricaux et gynécologiques. Ils avaient tous bénéficié d’une formation en technologie contraceptive. Ils ont été recrutés hors de la zone d’intervention du projet. Avant la collecte des données, une formation de deux journées leur a été accordée. Le deuxième jour de formation a été réservé au pré-test des outils. Un guide de l’enquêteur donnant des indications sur l’attitude à adopter pendant l’enquête, le mode de remplissage des outils ainsi que la définition des différents concepts utilisés a été élaboré et mis à la disposition des enquêteurs. Pendant la collecte des données sur le terrain, un contrôle de la qualité a été assuré à travers un suivi des équipes par deux superviseurs. Un ordre de mission a été remis aux binômes afin de leur permettre de marquer leur passage dans les centres de santé. Chaque binôme d’enquêteurs a réalisé la collecte de données dans deux centres de santé par jour. Les données qualitatives quant à elles ont été récoltées lors des entretiens individuels avec les bénéficiaires. Ces entretiens ont été réalisés en langue locale (dioula ou mooré) à l’aide du guide d’entretien, par une infirmière spécialisée en pédiatrie et expérimentée dans l’enquête qualitative. Les informations ont été enregistrées à l’aide d’un appareil enregistreur et retranscrites en français avant d’être analysées. Quatre entretiens qualitatifs ont été réalisés par jour. Les entretiens ont été réalisés hors du centre de santé et en l’absence des délégataires.

### Définitions des termes

Les termes suivants ont été utilisés dans cet article:

**Délégataire des prestations de service PF:** c’est un agent de niveau inférieur à qui l’on a transféré les compétences en matière de prestation d’une méthode contraceptive moderne donnée. Ils sont deux catégories ici: (1) les agents de première ligne (APL) qui sont constitués des IB, des AB, des AIS, et des AA; (2) les agents de santé communautaire (ASC).

**Délégant des prestations de service PF:** un agent de santé qui transfère les compétences et les attributions d’une prestation de services PF à un prestataire qui n’en était pas habilité. C’est l’infirmier d’Etat et la sage-femme ont été considérés comme délégants dans notre étude. Le mot sage-femme désigne aussi bien la sage-femme “homme“ appelé maïeuticien au Burkina Faso que la sage-femme “femme“.

**Nouvelle utilisatrice PF:** toute femme qui adhère pour la toute 1ère fois de sa vie à la PF quel que soit la méthode choisie [18].

**Ancienne utilisatrice:** toute femme qui, ayant déjà adhéré à la PF et arrêté pour une raison ou une autre (grossesse, abandon…) revient pour une méthode contraceptive [18].

**Qualité:** elle a été appréciée du point de vue du respect des recommandations médicales et le standard de soins à délivrer à la cliente. Elle a pris en compte les compétences techniques des prestataires, l’existence d’une gamme variée de méthodes et l’organisation des services PF ainsi que le coût des produits contraceptifs et des consommables médicaux. Elle n’a pas pris en compte la satisfaction des clientes.

### Les variables d’étude

Les variables suivantes ont été analysées:

**Sexe:** en deux modalités: homme et femme. Le sex ratio a été calculé selon la formule «homme/femme»; **Age:** en années dichotomisée en 2 classes: ≤ 34 et ≥ 35; **Etude:** en cinq catégories: non scolarisé, primaire, secondaire, BAC et BAC+; **Formation en PF:** en deux modalités de réponse: oui ou non; **Expérience professionnelle:** exprimée en nombre mois de service et catégorisée en trois modalités: ≤ 12 mois; 13 à 24 mois et > 24 mois; **Gestité:** en trois catégories: 0 grossesses, 2 à 5 grossesses et > 5 grossesses; **Parité:** en trois catégories: 0 enfant, 2 à 5 enfants et > 5 enfants; **Score de qualité des prestations:** en trois catégories: très satisfaisante si le prestataire était capable de pratiquer plus de 85% des éléments exigés par la norme satisfaisante si le prestataire était capable de pratiquer entre 60% et 85% des éléments exigés par la norme; faible s’il a été capable de pratiquer moins de 60% des éléments. Le score a été calculé pour les variables suivantes: accueil et counseling, déterminer le statut nouvelle ou ancienne utilisatrice, application des éléments contrôle et prévention des infections (PCI), connaissance des critères d’éligibilité aux méthodes contraceptives, connaissance de la réalisation des bilans de la cliente, connaissance des effets secondaires, insertion et retrait.

Pour l’évaluation de la qualité globale des services de PF offerts par les centres de santé, il a été opéré une analyse suivant les six piliers de l’OMS [15]: **Gouvernance** regroupant: l’organisation des services de PF, la recherche des perdus de vu, l’intégration de la PF dans les autres services, la disponibilité d’un protocole de prise en charge des accidents d’exposition au sang et produits biologiques (AES) et la permanence du service PF. **Prestation** regroupant l’offre des méthodes contraceptives (accueil, counseling, bilan, administration du produit), la gestion des déchets biomédicaux, la disponibilité d’un incinérateur fonctionnel et la disponibilité des bacs pour décontamination. **Approvisionnement** regroupant: la disponibilité des équipements et la disponibilité des produits contraceptifs. **Système d’information sanitaire** qui regroupe: la disponibilité des documents, normes et outils de surveillance, la collecte et la transmission des données et l’archivage des documents. **Financement** qui regroupe: l’application des prix de vente et le respect du circuit d’approvisionnement. **Ressource humaine** qui regroupe: le respect des normes en personnel, la formation et la supervision.

### Analyse des données

Les données quantitatives collectées ont été saisies dans un masque de saisie élaboré sur le logiciel Epi info 7 avant leur analyse selon un plan d’analyse des données. Avant de procéder à l’analyse proprement dite, un travail de nettoyage des données a été effectué afin d’avoir une base de données propre devant servir à l’extraction des résultats. Cette opération a consisté au contrôle et à la correction des erreurs commises lors de la collecte telles que les incohérences, les omissions et les valeurs aberrantes. Elle a permis de vérifier la cohérence interne des réponses questionnaire après questionnaire. L’analyse univariée a permis de mesurer les caractéristiques de tendances centrales et de dispersion pour les variables quantitatives. Elle a permis également de déterminer la fréquence des différentes modalités de variables afin de pouvoir détecter les valeurs aberrantes ainsi que le taux de non réponse pour les différentes questions. L’analyse bivariée quant à elle a permis de déterminer les valeurs des indicateurs désagrégés par type de prestataire (Délégant vs délégataire). Le test du Chi carré a été utilisé pour les variables catégorielles, et le test t de Student pour les variables quantitatives afin de tester s’il y a une différence significative entre les résultats des délégants et les délégataires en matière de qualité d’offre des méthodes contraceptives. Dans chacun de ces deux cas, un seuil de signification de 5% a été utilisé pour tirer la conclusion du test. L’analyse multivariée a été également réalisée afin d’éliminer des éventuels biais de confusion. Pour le niveau de compétence, une analyse comparative de celui des délégataires médicaux a été réalisée avec celui des sages-femmes et des infirmiers d’Etat travaillants dans le même contexte, dans les centres de santé de la zone d’intervention du projet DT. Pour la détermination du score de qualité des services PF délivrés par le délégataire, il a été réalisé une combinaison des scores obtenus pendant l’observation de l’offre des méthodes contraceptives et ceux obtenus pendant l’examen des connaissances réalisés à l’aide de scénarios de cas et de questions à choix multiples. Pour l’analyse des données qualitatives, il a été fait usage de la technique d’analyse du contenu. L’information obtenue à lors des entretiens a été triangulée avec celle obtenue à travers les méthodes quantitatives. Par ailleurs, une analyse préliminaire des données qualitatives a été faite tout au long de la période de collecte des données (au cours des séances quotidiennes de débriefing des enquêteurs et au fur et à mesure que les transcriptions étaient disponibles) afin de pouvoir incorporer des questions supplémentaires sur des thèmes émergents.

## Considérations éthiques

Le premier principal enjeu éthique de cette étude réside dans le respect des répondants qui ont été sollicités pour confier des informations. C’est pourquoi, les objectifs de l’enquête ont été préalablement décrits par les enquêteurs aux enquêtés avant de procéder à l’entretien. Ces derniers avaient le loisir d’accepter ou de refuser de répondre aux questions. Pour ceux qui ont accepté, un consentement libre et éclairé a été signé. Ils avaient le droit de ne pas répondre aux questions sans devoir donner une motivation à ce refus. Lors des observations de l’offre des méthodes contraceptives, les enquêteurs portaient la blouse blanche afin de rassurer les patientes. Le deuxième principal enjeu éthique de cette recherche réside au niveau de la délicatesse du sujet de la contraception dans notre contexte et en particulier lors de l’identification, la recherche et l’entretien avec les femmes ayant bénéficié des méthodes contraceptives. Pour réduire ce risque et conserver leur anonymat, les enquêteurs ont été formés au préalable sur ces enjeux et le respect des consignes qui leur ont été données. Pour l’enjeu éthique lié à la manipulation des données pour des aspects liés à la confidentialité, les données collectées ont été rendues anonymes et la confidentialité a été sauvegardée. Avant le démarrage de l’étude, une autorisation d’enquête a été fournie par le directeur régional de la santé de la Boucle du Mouhoun.

## Résultats

Tous les centres de santé (20), ainsi que tous agents de santé (APL, SFE et IDE) de la zone d’intervention du projet DT du district sanitaire de Tougan ont été inclus dans l’étude. Sur ces 20 centres de santé, dix (10) disposaient d’un délégant dont cinq (5) d’une sage-femme.

**Caractéristiques socio-professionnelles des délégataires et des délégants enquêtés:** le Tableau 1 présente les caractéristiques socio-professionnelles des délégataires (APL et ASBC) et celles des délégants (SFE et IDE). L’étude a porté sur 35 ASBC et 54 agents de santé constitués de 35 APL et 19 SFE/IDE. Ils étaient majoritairement jeunes. En effet, plus de la moitié des enquêtés avait moins de 34 ans. Leur âge moyen était de 34 ans (ET= ±5,3) pour les APL; 34,7 (ET= ±6,8) pour les délégants et 34 ans (ET= ±7,0) pour les ASBC. Le sex ratio de l’ensemble des prestataires était de 1,5 en faveur des hommes. Il était de 1,1 pour les APL; 2,1 pour les ASC et 1,3 pour les délégants. Si les APL et les délégants avaient en majorité le niveau secondaire (63% et 42%), plus de deux tiers des ASC avaient le niveau primaire (74,2%). En plus, cinq (5) délégants avaient le niveau BAC+, tandis qu’un des ASC n’était pas scolarisé. Aussi, plus de deux tiers des APL et des délégants avaient une expérience professionnelle de plus de deux ans tandis que 84% des ASC avaient une expérience de moins de deux ans (Tableau 1).

**Tableau 1:** caractéristiques socio-professionnelles des délégataires et des délégants enquêtés dans le district sanitaire de Tougan en décembre 2017

Variables	Délégataires				Délégants	
	APL (IB, AB, AIS, AA)		ASC		SFE et IDE	
	n	%	n	%	n	%
Sexe						
**F**	17	48,6	11	31,4	8	42,1
**M**	18	51,4	24	68,6	11	57,9
Age **(année)**						
**≤ 34**	19	54,2	19	54,2	12	63,2
**≥ 35**	16	45,8	16	45,8	7	36,8
Etude						
**Non scolarisé**	0	-	1	2,9	0	-
**Primaire**	13	37.1	26	74,2	1	5,3
**Secondaire**	22	62.9	7	20,0	9	47,4
**BAC**	0	-	1	2,9	4	21,0
**BAC+**	0	-	0	-	5	26,3
Formation en PF						
**Oui**	25	71,4	35	100.0	16	84,2
**Non**	10	28,6	0	-	3	15,8
Expérience prof **(mois)**						
**≤ 12**	4	11,4	13	42,0	3	20,0
**13-24**	10	28,6	13	42,0	2	13,3
**>24**	21	60,0	5	16,0	10	66,7

**Caractéristiques socio-démographiques des utilisatrices enquêtées:** dix-neuf (19) utilisatrices ayant bénéficié de méthodes contraceptives modernes auprès des délégataires ont été interviewées: 11 auprès des ASC et 8 auprès des APL. Neuf (9) des bénéficiaires étaient sous une méthode de longue durée d’action (cinq sous implants et quatre sous DIU), six sous une méthode injectable et quatre sous une contraceptive orale. Cinq d’entre elles habitaient dans un village situé à plus de 5 km d’un centre de santé. Quant à leur profession, 18 étaient des ménagères, une était élève et une autre était institutrice. Leur âge moyen était de 33,7 ans (ET=± 6,3). Pour la gestité, deux (2) bénéficiaires étaient nulligestes, neuf (9) avaient déjà eu entre deux et cinq grossesses, huit (8) en avaient eu plus de cinq. Quant au nombre d’enfants, trois (3) des bénéficiaires n’avaient aucun enfant tandis que sept (7) avaient plus de cinq enfants. Selon les registres de planification familiale des centres de santé, 11 des bénéficiaires étaient des nouvelles utilisatrices des méthodes contraceptives modernes.

**Qualité de l’accueil/counseling, la détermination du statut nouvelle ou ancienne utilisatrice et le contrôle et prévention des infections:** comme le montre le Tableau 2, l’accueil et le counseling ont été évalués ensemble. Le score de qualité a été classée satisfaisante aussi bien chez les APL (82%) que chez les ASC (75,8%). Il existait une différence statistiquement significative entre le score de qualité des ASC et celui des délégants (P=0,014) en matière d’accueil et du counseling. Par contre, pour la détermination du statut de nouvelle ou d’ancienne utilisatrice de méthode contraceptive et pour le contrôle et la prévention des infections, il n’existait pas de différence statistiquement significative entre le score de qualité des délégataires et celui des délégants Il ressort des entretiens réalisés auprès des utilisatrices, que quatre (4) erreurs ont été commises par les délégataires dans la détermination du statut de nouvelle ou d’ancienne utilisatrice soit une erreur sur cinq (20%). De ces quatre erreurs, trois (3) ont été commises par les ASC. Quant au contrôle et à la prévention des infections, toutes les utilisatrices ont noté que les délégataires ont lavé leur main avant et après l’administration du produit contraceptif aussi bien au centre de santé qu’en communauté. Les ASC disposaient en effet d’un dispositif de lavage des mains dans leur domicile. De même, les aiguilles usagées ont été jetées dans des boites à tranchant aussi bien en communauté qu’au centre de santé. Une fois rempli, la boite à tranchant de l’ASC devrait être ramenée au centre de santé. Seulement, aucune des boites à tranchant des ASC n’était encore depuis le démarrage du projet. Quant aux informations données aux utilisatrices lors du counseling, seules deux (2) utilisatrices sur les 19 enquêtées ont affirmé avoir reçu des informations sur le VIH et les infections sexuellement transmissibles. Du point de vue des bénéficiaires sur l’accueil et en particulier sur la discrétion observée, les deux (2) récits ci-dessous recueillis dans deux villages différents présentent deux pratiques différentes mais révélatrices de l’effort fournis par les délégataires pour protéger les utilisatrices des méthodes contraceptives. Récit 1: «*Quand je suis arrivée, je les ai salués et on m’a donné la place pour m’asseoir avant de commencer la causerie. Ici comme certains hommes n’acceptent pas et que les femmes se cachent, on ne nous aligne pas, nous allons une à une» (femme de 34 ans sous pilule*). Récit 2: «*Quand nous sommes arrivées, on nous a dit de nous aligner avec les malades et d’attendre notre tour avant de rentrer. Si on nous séparait cela n’était pas bien parce-que tout le monde allait se rendre compte de ce pourquoi nous sommes là, mais de cette manière c’est une fois dans la salle de consultation qu’on explique le motif de notre venue à l’agent de santé» (femme de 45 ans sous DIU)*.

**Tableau 2:** qualité de l’accueil/counseling, la détermination du statut de la cliente et la prévention et le contrôle de l’infection par le délégataire dans le district sanitaire de Tougan en décembre 2017

Variables	Délégants		Délégataires					
	SFE et IDE		APL (IB, AB, AIS, AA)			ASC		
	n	Score qualité	n	Score qualité (%)	P-valeur	n	Score qualité (%)	P-valeur
**Accueil et counseling**	16	87,5	24	82,1	0,16	36	75,8	˂0.05
**Détermination statut nouvelle ou ancienne utilisatrice**	14	92,9	24	100,0	-	36	96,6	0.345
**Contrôle et prévention des infections**	14	92,9	24	92,7	0,39	32	97,1	0.098

**Qualité de l’offre des implants et du DIU:** le Tableau 3 présente les scores de qualité des délégataires en matière d’offre des implants et des DIU. Le score de qualité des délégataires pour l’offre des implants était de 79,2% dans le domaine de la maitrise des critères d’éligibilité de la cliente; 70,8% au niveau de l’insertion et le retrait de l’implant et 63% au niveau de la connaissance des effets secondaires des produits contraceptifs. Dans ces trois domaines, la qualité des prestations des délégataires a été jugée satisfaisante. Cependant, il n’existait une différence statistiquement significative qu’au niveau de la maitrise des critères d’éligibilité de l’implant et des effets secondaires où la qualité de la prestation des délégataires semblait meilleure à celle des délégants. Par contre au niveau du bilan de la cliente, la qualité des délégataires a été classée faible sans toutefois qu’il existe une différence statistiquement significative entre le score de qualité des délégataires et celui du délégant. Au niveau de la technique de l’offre du DIU, le score de qualité des délégataires a été globalement classé satisfaisant au niveau des quatre domaines qui ont été examinés. Aussi, avec un score de 63,6%, la qualité de la prestation des délégataires semblait meilleure à celle des délégants au niveau de la maitrise des effets secondaires du DIU. A ce niveau Il existait une différence statistiquement significative entre le score de qualité des délégataires avec celui des délégants.

**Tableau 3:** qualité de l’offre des implants et du DIU par le délégataire dans le district sanitaire de Tougan en décembre 2017

Variables	Délégants		Délégataires		
	SFE et IDE		APL (IB, AB, AIS, AA)		
	n	Score de qualité (%)	n	Score de qualité (%)	P-valeur
Offre des implants					
**Critères d’éligibilité**	14	64,3	24	79,2	˂0.05
**Bilan de la cliente**	14	61,5	22	59,1	0.35
**Insertion et retrait**	14	78,6	24	70,8	0,096
**Effets secondaires**	18	50,0	24	63,0	˂0.05
Offre des DIU					
**Critères d’éligibilité**	14	81,3	23	73,9	0.155
**Bilan de la cliente**	16	68,8	22	68,2	0.439
**Insertion et retrait**	16	81,3	22	81,8	0,428
**Effets secondaires**	16	50,0	22	63,6	˂0.05

**Qualité de l’offre des contraceptives injectables et orales:** Bien qu’il n’existât pas une différence statistiquement significative avec le délégant, le score de qualité des délégataires a été classé faible au niveau de la maitrise du bilan de la cliente désirant une méthode contraceptive injectable (Tableau 4). Paradoxalement, au niveau de l’offre de la pilule, il a été observé une différence statistiquement très significative entre le score de qualité des ASC et celui des délégants dans le domaine de la réalisation du bilan clinique chez la cliente désireuse d’une méthode contraceptive injectable. De surcroit la qualité du bilan des ASC (88,2%) semblait meilleure à celle des délégants. D’une manière générale, l’élément qui était le moins bien maitrisé dans la réalisation du bilan de la cliente était la détermination d’une éventuelle grossesse. Comme l’a rapporté une utilisatrice de pilule à qui l’on a demandé si elle avait été examinée lors de sa consultation, les prestataires appliquent insuffisamment les critères de l’OMS qui permettent de s’assurer raisonnablement que la cliente n’est pas enceinte. «*Oui, on regarde si tu es enceinte, parce qu’il faut que tu sois en règles avant qu’on te donne le produit pour cela, on t’installe et on t’examine. Si c’est avant le 42^e^jour après ton accouchement, on te demande si tu n’as pas eu de rapports sexuels avec ton mari» (femme de 34 ans sous pilule)*. Outre le bilan de la cliente, l’on observe une différence statistiquement significative entre le score de qualité des ASC (50%) et celui des délégants (69,2) du point de vue la maitrise de la technique d’injection du produit contraceptif (P ˂0,001). Il en est de même pour la maitrise des effets secondaires où le score de qualité des ASC était de 69,3% (P ˂0,05).

**Tableau 4:** qualité de l’offre des injectables et pilules par le délégataire dans le district sanitaire de Tougan en décembre 2017

Variables	Délégants		Délégataires					
	SFE et IDE		APL (IB, AB, AIS, AA)			ASC		
	n	Score qualité (%)	n	Score qualité (%)	P-valeur	n	Score qualité (%)	P-valeur
Offre des injectables								
Critères d’éligibilité	15	73,3	24	75,0	0.419	36	63,9	0,085
Bilan de la cliente	16	62,5	24	58,3	0.252	32	59,4	0,331
Injection	16	75,0	26	69,2	0,173	36	50,0	˂0,001
Effets secondaires	16	50,0	24	62,5	0,036	33	69,3	˂0,05
Offre des contraceptifs oraux								
Critères d’éligibilité	16	62,5	23	73,9	0.039	23	74,0	0,038
Bilan de la cliente	17	64,7	23	69,6	0.226	34	88,2	˂0,001
Offre des pilules	16	68,8	23	69,6	0,439	34	76,5	0,125
Effets secondaires	16	75,0	23	69,6	0.215	35	57,1	˂0,05

**Qualité globale des services de PF dans les centres de santé:** le Tableau 5 présente le score de qualité globale des 20 centres de santé de la zone d’intervention du projet délégation des tâches par pilier de l’OMS. Ce score était de 72,8% pour l’ensemble des 20 centres de santé. La qualité de deux piliers a été classée très satisfaisante. Il s’agit du financement (95%) et l’approvisionnement en produits contraceptifs et autres consommables (87,5%). Les plus faibles scores ont été observés au niveau des piliers prestations des services (67,7%), la gouvernance (69,2%) et la ressource humaine (79,5%). Des entretiens réalisés avec les utilisatrices, le prix des produits contraceptifs ainsi que les directives relatives à la gratuité des consommables ont été respectés chez 17 des 19 bénéficiaires. Les deux cas de non-respect des prix concernent l’offre de l’implant et de l’injectable. La 1ère a été offerte à 5,6 dollar US au lieu de 0,93 dollar US. Quant à la seconde, elle a été offerte à 2,8 dollar US au lieu de 0,47 dollar US. Par ailleurs, les utilisatrices qui ont eu recourt aux services PF pendant la semaine de nationale de la PF organisée par le ministère de la santé avec l’appui du fonds des nations unies pour la population ont bénéficié gratuitement des méthodes contraceptives comme l’indique le récit ci-dessous. «*Pour cette fois-ci je n’ai rien payé mais pour la fois passée j’avais dépensé 2500 frs pour placer et 1500 frs pour le retrait*«(femme de 39 ans ayant bénéficié de l’implant pendant la semaine nationale de la PF). De même, contrairement aux directives, les consommables auraient été vendus à certaines clientes. «Quand j’ai décidé de placer le DIU… ils m’ont remis une ordonnance de compresses et je suis allée chercher. De retour, ils m’ont installé et nettoyé mon vagin avec un produit puis ils ont placé le DIU» (femme de 43 ans sous DIU). Comme le rapporte les bénéficiaires, les services de PF semblaient intégrés au système local de santé. Les délégataires profiteraient des autres demandes de soins pour parler de la contraception. «*C’est lors des pesées de mes enfants qu’ils nous ont parlé de la planification familiale. Ils nous ont dit de bien nous occuper des enfants et si on veut leur donner à manger, de laver les plats sinon si les assiettes sont sales les enfants feront la diarrhée. Par rapport à la planification familiale, ils nous ont dit de faire la contraception afin d’espacer les naissances entre 2 à 3 ans ou 5ans pour pouvoir nous reposer* » (femme de 34 ans sous méthode injectable). «Au CSPS lorsque tu accouches, les agents de santé te disent de faire la contraception afin que ton enfant grandisse avant de prendre une nouvelle grossesse» (femme de 26 ans sous méthode injectable). Toutefois, les entretiens ont révélé également que certaines clientes auraient bénéficié d’une méthode contraceptive sans être passées préalablement par un counseling individuel. A la question de savoir s’il y avait eu un entretien avant l’administration du produit contraceptif, l’on a recueilli cette réponse: «*Non, après la sensibilisation il n’y a plus eu d’entretien au centre de santé. C’est lors de la sensibilisation avec les autres femmes qu’on nous a parlé de la PF*«(femme de 24 ans sous implant). Aussi, à la question de savoir si l’utilisatrice avait pu poser des questions d’éclaircissements, cette réponse a été donnée: «*non, parce que comme nous étions nombreuses dans la salle (on valait 10 personnes), j’avais honte de demander certaines choses*«(femme de 39 ans sous implant). En dehors des effets secondaires à type de vertige, prise de poids ou trouble du cycle menstruel qui ont été pris en charge par les prestataires, aucune participante à l’enquête n’a signalé un incident en lien avec la maitrise de l’administration du produit contraceptif. «*J’avais des saignements qui étaient abondants. Je suis repartit au centre de santé leur expliquer, ils m’ont prescrit un produit et les saignements se sont arrêtés*«(femme de 34 ans sous DIU).

**Tableau 5:** qualité des services de PF offerts dans les centres de santé de la zone d’intervention du district sanitaire de Tougan en décembre 2017

Piliers	Variables	n	Score qualité (%)	
Gouvernance	Organisation des services	20	70,0	69,2
	Recherche de perdus de vu	20	55,0	
	Intégration de la PF dans le paquet minimum	20	90,0	
	Disponibilité des protocoles de prise en charge des accidents d’exposition au sang et produits biologiques	20	60,0	
	Permanence service PF	20	80,0	
Prestation	Offre des méthodes contraceptives	38	66,2	67,7
	Gestion des déchets biomédicaux satisfaisant	20	75,0	
	Disponibilité incinérateur fonctionnel	20	85,0	
	Disponibilité bacs pour décontamination	20	60,0	
Approvisionnement	Disponibilité équipements d’insertion/retrait	20	100,0	87,5
	Disponibilité des PC aux cours 3 derniers	20	75,0	
Système d’information sanitaire	Disponibilité des documents, normes et outils de surveillance	20	70,0	
	Rapportage et archivage	20	95,0	
Financement	Application des prix de vente et circuit d’approvisionnement	20	95,0	95,0
Ressource humaine	Respect normes en personnel	20	75,0	79,5
	Formation en PF clinique	38	76,3	
	Supervision	20	90,0	

## Discussion

La présente étude a eu pour objectif de déterminer la qualité des services de planification familiale offerts par le délégataire dans le contexte de la mise en œuvre du projet d’expérimentation du transfert des compétences dans le domaine de la PF. Le délégataire était représenté ici par les APL et les agents de santé communautaires. Les méthodes contraceptives modernes testées étaient le DIU, les implants, les injectables et les pilules. Des expériences similaires d’implémentation de la délégation des tâches dans le domaine de la PF ont été réalisées en Ethiopie, au Sénégal, en Tanzanie et en Inde avec l’Implanon, la ligature des trompes et le DIU du post-partum [10-12, 19]. Selon le document de politique et normes en matière de santé de la reproduction du Burkina Faso, les APL ne sont pas autorisés à offrir les implants ou le DIU. De même, le personnel communautaire n’est pas habilité à offrir les contraceptifs injectables ou les pilules [10]. Bien que l’OMS recommande l’extension de l’offre des méthodes contraceptives de longue durée d’action au personnel de niveau inférieure ainsi que celle des pilules et des injectables aux agents de santé communautaire [1], la situation du Burkina Faso n’est guère singulière.

En effet, malgré la mise en œuvre de projets pilotes dans certains pays (Togo, Benin, Sénégal, Mauritanie), les documents de politique et normes en matière de santé de la reproduction de nombreux pays africains restreignent l’offre des méthodes contraceptives de longues durées d’action aux sages-femmes, aux infirmiers d’Etat et aux médecins [2-5]. Dans ce contexte africain, de pénurie de la ressource humaine qualifiée en milieu rural, cette restriction réduit la couverture géographique de l’offre des méthodes contraceptives concernées [1]. A titre d’exemple, dans cette étude qui porte sur 20 centres de santé parmi lesquels seuls 10 disposaient d’un délégant dont cinq d’une sage-femme, les méthodes contraceptives de longue durée d’action ne seraient offertes que dans 50% de ces centres de santé uniquement. Cela corrobore les résultats de l’enquête nationale sur la disponibilité des médicaments vitaux de santé maternelle réalisée au Burkina Faso en 2016, qui rapportait que 41,4% des centres de santé périphériques du Burkina Faso ne disposaient pas de personnels capables de fournir le DIU. Ce chiffre était de 33% pour les implants [20]. A ce propos, l’OMS indiquait en 2013, qu’il manquait environ 7,2 millions de sages-femmes, d’infirmières et de médecins pour atteindre un seuil d’à peine 34,5 professionnels de santé qualifiés pour 10 000 habitants. Sur la base de ce constat, l’OMS notait que «les modèles verticaux fondés sur les établissements de santé, tributaires des médecins …ne sont ni optimaux ni durables. La qualité des soins peut être assurée par une nouvelle répartition des tâches» [21]. Outre le problème de couverture géographique, la restriction de l’offre des méthodes contraceptives aux sages-femmes, aux infirmiers d’Etat et aux médecins réduit la disponibilité des méthodes contraceptives dans les centres de santé dépourvus de personnel qualifié capable de les offrir. Effet, selon les résultats de l’enquête nationale sur la disponibilité des produits contraceptifs réalisée en 2016 au Burkina Faso, seules un quart (25,2%) des centres de santé ont dans leur pratique, l’offre du DIU [20]. Il est de ce fait, loisible de penser que dans 75% des centres de santé du Burkina Faso (qui ne disposent pas de DIU), le principe du choix volontaire et indépendant des méthodes contraceptives par les utilisatrices est gravement entamé. Il en est de même pour ce qui est de l’accessibilité et de l’adhésion des femmes à ces méthodes non disponibles. Du reste dans sa publication sous la forme d’aide-mémoire N°351 du mois de février 2018, l’OMS déclarait que le “choix limité des méthodes contraceptives“ constitue un des facteurs qui explique qu’en Afrique, 23,5% des femmes en âge de procréer ont un besoin non satisfait de moyens de contraception modernes [22].

Nonobstant ces constats et bien que la délégation de tâches dans le domaine de la PF contribue à améliorer la couverture géographique des méthodes contraceptives ainsi que le respect du droit de la femme, il est indispensable de maintenir un niveau de qualité et de sécurité des services fournis aux bénéficiaires irréprochable lors de sa mise en œuvre [1]. Du reste, à la question de savoir si ce transfert de compétence était possible dans notre contexte, un gynécologue obstétricien du Burkina qui a été interviewé dans le cadre d’une étude réalisée par Gandaho et collaborateurs en 2014, disait ceci: »Si nous avons délégué l’accouchement, qui comporte plus de risque pour une femme, à une accoucheuse en lieu et place de la sage-femme, nous devons logiquement autoriser cette accoucheuse à insérer le DIU ou l’implant. Ce geste est plus simple et moins risqué» [4]. La présente étude se justifie donc par le besoin d’apprécier le niveau de qualité des services de PF offerts par le délégataire dans le contexte particulier de la mise en œuvre du projet pilote de la délégation des tâches implémenté par le ministère de la santé burkinabè avec l’appui de l’ONG Marie Stopes Burkina, l’association burkinabè pour le bien-être familial et Equilibre et population. Bien que les expériences pilotes de délégation des tâches dans le domaine de la PF soient nombreuses en Afrique, les études similaires sur l’examen de la qualité des services PF associés au transfert des compétences restent rares dans la littérature [11, 23]. Aussi cette étude a-t-elle permis d’examiner l’offre des soins contraceptifs de 35 agents de santé communautaires et 54 prestataires de soins (dont 35 APL et 19 sages-femmes et infirmiers d’Etat) de 20 centres de santé.

Des résultats, il est ressorti que le score de qualité global des services de PF fournis dans les centres de santé de la zone d’intervention du projet délégation des tâches était de 72,8%. Le score de qualité des délégataires pour l’offre des implants était de 70,8% au niveau de l’insertion et le retrait de l’implant. En Ethiopie, Nuccio et collaborateurs ont relevé un score moyen de respect des standards et protocoles de 96,9% chez le délégataire [12]. La différence observée au niveau des deux performances pourrait être liée à deux facteurs principaux: la différence des outils utilisés pour les deux études ainsi que la différence du contexte. En effet, l’intervention que nous avons évaluée s’étend sur une vingtaine de centres de santé publiques ayant un paquet d’activités incluant l’offre de plusieurs autres services alors que l’étude d’Ethiopie concerne des prestataires privés d’une ONG qui ne font qu’offrir les services de PF. Quant à la maitrise des effets secondaires du DIU, de leur explication à la cliente ainsi que la prise de mesures correctrices, le score de qualité des délégataires était satisfaisant (63,6%). De même aucune incidence majeure n’a été notée chez le délégataire. Ce résultat est corroboré par l’étude réalisée par Vivek et collaborateurs en Inde qui ont trouvé également qu’il n’y avait pas de différence statistiquement significative entre la survenue d’effets secondaires ou d’incidents chez le délégataire (infirmier) et le délégant (médecin). Barone et collaborateurs ont abouti à la même conclusion dans le cadre de la délégation des tâches dans le domaine de la ligature des trompes en Tanzanie [24]. Aussi, l’offre de la contraception orale par les agents de santé communautaire a été jugée satisfaisante dans les domaines tels que l’accueil, le counseling, le contrôle et la prévention des infections. Par contre, le score de qualité de l’ASC a été classé faible au niveau de la maitrise de la technique l’injection (50%). Si cette situation peut s’expliquer par l’effet induit par la présence de l’enquêteur sur l’ASC au moment de la collecte des données, il pourrait également révéler une insuffisance dans la formation de ces ASC. En effet, cette formation n’a duré que dix jours. Elle pourrait être liée également à leur expérience sur cette pratique des injections qui reste relativement courte (8 mois au moment de la collecte des données). Pour Folz et Ali (Afghanistan, Égypte et Pakistan), les facteurs qui influencent négativement la qualité des services de santé communautaires sont: la sécurité au niveau de l’approvisionnement des produits, le caractère inadéquat et irrégulier des formations et le système de rémunération imprévisible ou inadéquat [25].

Toutefois ce constat nous renvoie au postulat que la délégation de l’offre des méthodes injectables aux ASC pourrait être classée dans la catégorie 2 des recommandations du cadre DECIDE de l’OMS. Cette catégorie indique qu’une telle intervention doit s’accompagner d’une surveillance et d’une évaluation rigoureusement ciblée [21]. L’analyse de la qualité globale des services de PF offerts dans les 20 centres de santé de la zone d’intervention indique un score globalement satisfaisant (72,8%) avec des variations indexant les piliers gouvernance, prestation des services et la ressource humaine comme des domaines qui requièrent une plus grande attention. Ce constat a été noté également par Rodriguez et collaborateurs en 2014 [23]. En dépit des résultats observés par la présente étude utilisant l’observation en temps réel des prestations de soins, des entretiens et la revue comme techniques de collecte, il a pu être relevé des limites. En effet, Il est possible que l’influence de l’enquêteur sur le prestataire et la cliente ait induit des erreurs. Il est à craindre également, un biais de confusion pouvant être lié à l'interférence de variables qui n’ont pas été prises en compte dans l’analyse des données. Aussi, des limites en lien avec le type d’outils de collectes utilisés ainsi que des biais de mémoire pourraient entacher les résultats de cette étude. Du reste, bien que le nombre de délégants (19) soit inférieur à celui des délégataires (35 ASBC et 35 APL), les résultats obtenus chez chacun des deux catégories de prestataires restent comparables dans la mesure où la collecte des données a été réalisée dans les conditions et contexte identiques. En plus, les délégants et les délégataires, en particulier les APL et délégants avaient des caractéristiques socio-professionnelles très similaires. Majoritairement jeunes, Ils avaient tous un âge moyen de 34 ans. Les prestataires masculins étaient plus nombreux aussi bien chez les délégataires que les délégants avec un sex ration de 1,1 pour les APL et 1,3 pour les délégants. Soixante-trois pour cent (63%) des APL avaient le niveau d’étude secondaire contre 42% des délégants. Quant aux ASBC, ils avaient majoritairement le niveau d’étude primaire (74,2%) et seulement 20% d’entre eux avaient le niveau secondaire. Bien que le niveau de scolarisation des ASBC de cette étude soit inférieur à celui des APL et des délégants enquêtés, il constitue une évolution exceptionnelle dans le système de santé burkinabè et reflète les reformes entreprises ces dernières années par le gouvernement burkinabè pour améliorer les soins communautaires. En effet en 2014, selon une étude réalisée par l’Institut Supérieur des Sciences de la Population au Burkina dans 9 districts sanitaires du Burkina Faso 45% des ASC étaient non scolarisés tandis que 39% avaient le niveau primaire [26].

## Conclusion

Dans le contexte de rareté de la ressource humaine qualifiée, la restriction de l’offre des méthodes contraceptives de longue durée d’action aux sages-femmes, aux médecins et infirmiers d’Etat comme le stipule le document de politiques et normes en matière de santé de la reproduction de la plupart de nos pays aujourd’hui, réduit fortement l’accessibilité et l’adhésion des femmes à ces méthodes. Cette restriction remet également en cause le principe du choix volontaire et indépendant de la méthode contraceptive par les utilisatrices. L’expérience de délégation des tâches dans le domaine de la planification familiale telle que développée dans le district sanitaire de Tougan à l’avantage d’améliorer la couverture géographique de l’offre des méthodes contraceptives de longue durée d’action ainsi que celle des méthodes contraceptives injectables. Elle améliore également le respect du droit de la femme dans la mesure où elle offre à l’utilisatrice la possibilité de choisir librement et volontairement la méthode contraceptive de son choix parmi une gamme plus variée de produits contraceptifs. Toutefois, sa mise en œuvre recommande de maintenir un niveau de qualité et de sécurité des services PF irréprochable comme le recommande l’OMS. Nonobstant les limites de cette étude, elle nous donne une idée de la qualité des services PF offerts ainsi que la capacité des agents de niveau inférieur à fournir les méthodes contraceptives dont l’offre ne leur étaient pas autorisées. Sous certaines conditions (renforcement des compétences, suivi, coaching), il est bien possible d’étendre l’offre des méthodes contraceptives de longues durées aux APL ainsi que celle des injectables aux ASC tout en conservant un niveau de qualité des services PF satisfaisant.

### Etat des connaissances actuelles sur le sujet

- Bien que l’Organisation Mondiale de la Santé recommande le transfert des compétences de l’offre des méthodes contraceptives aux agents de santé de niveau inférieur, les documents de politique et normes en matière de santé de la reproduction de nombreux pays africains restreignent l’offre des méthodes contraceptives de longues durées d’action aux sages-femmes et aux médecins;
- Pourtant, du fait de la pénurie en personnel qualifié, en l’occurrence en médecins et en sages-femmes dans le milieu rural africain, la restriction de l’offre des méthodes de longue durée d’action aux sages-femmes et aux médecins réduit fortement la couverture géographique de ces méthodes contraceptives et donc leur utilisation;
- De nombreuses expériences pilotes de délégation des tâches dans le domaine de la PF ont été implémentées avec des résultats encourageants en Afrique et en Asie.

### Contribution de notre étude à la connaissance

- Cette étude établie que la qualité des services PF offerts par le personnel de niveau inférieur peut être comparée à celle des sages-femmes et des médecins;
- Elle confirme que bien formés, bien suivis et coachés, le personnel de santé de niveau inférieur peut offrir les méthodes contraceptives de longue durée d’action avec une qualité irrépréhensible.
